# Increased Resistance of Bt Aspens to *Phratora vitellinae* (Coleoptera) Leads to Increased Plant Growth under Experimental Conditions

**DOI:** 10.1371/journal.pone.0030640

**Published:** 2012-01-24

**Authors:** Joakim Hjältén, E. Petter Axelsson, Thomas G. Whitham, Carri J. LeRoy, Riitta Julkunen-Tiitto, Anders Wennström, Gilles Pilate

**Affiliations:** 1 Department of Wildlife, Fish and Environmental Studies, Swedish University of Agricultural Science, Umeå, Sweden; 2 Department of Biological Sciences, Northern Arizona University, Flagstaff, Arizona, United States of America; 3 Environmental Studies Program, The Evergreen State College, Olympia, Washington, United States of America; 4 Department of Biology, University of Eastern Finland, Joensuu, Finland; 5 Department of Ecology and Environmental Science, Umeå University, Umeå, Sweden; 6 Génétique et Physiologie Forestières, INRA, UR0588 Amélioration, Orléans, France; University College Dublin, Ireland

## Abstract

One main aim with genetic modification (GM) of trees is to produce plants that are resistant to various types of pests. The effectiveness of GM-introduced toxins against specific pest species on trees has been shown in the laboratory. However, few attempts have been made to determine if the production of these toxins and reduced herbivory will translate into increased tree productivity. We established an experiment with two lines of potted aspens (*Populus tremula*×*Populus tremuloides*) which express Bt (*Bacillus thuringiensis*) toxins and the isogenic wildtype (Wt) in the lab. The goal was to explore how experimentally controlled levels of a targeted leaf beetle *Phratora vitellinae* (Coleoptera; Chrysomelidae) influenced leaf damage severity, leaf beetle performance and the growth of aspen. Four patterns emerged. Firstly, we found clear evidence that Bt toxins reduce leaf damage. The damage on the Bt lines was significantly lower than for the Wt line in high and low herbivory treatment, respectively. Secondly, Bt toxins had a significant negative effect on leaf beetle survival. Thirdly, the significant decrease in height of the Wt line with increasing herbivory and the relative increase in height of one of the Bt lines compared with the Wt line in the presence of herbivores suggest that this also might translate into increased biomass production of Bt trees. This realized benefit was context-dependent and is likely to be manifested only if herbivore pressure is sufficiently high. However, these herbivore induced patterns did not translate into significant affect on biomass, instead one Bt line overall produced less biomass than the Wt. Fourthly, compiled results suggest that the growth reduction in one Bt line as indicated here is likely due to events in the transformation process and that a hypothesized cost of producing Bt toxins is of subordinate significance.

## Introduction

The future challenges for forestry are demanding due to changes in climate and intensified land-use [1]. Fossil fuels will need to be replaced with renewable energy sources, which will affect not only agriculture practices, but also silviculture (tree production). Forests can, in theory, become a major source of bioenergy in the future and have the potential to mitigate the anticipated rise in CO_2_ over the next 50 years. However, this calls for improvements in tree characteristics as well as changes to management practices and technology [1], [2].

To facilitate such mitigation, genetic engineering is a useful compliment to other practices as it may partially alleviate some of the constraints on conventional tree breeding. Conventional tree breeding is based on natural variation in economically important traits. Forest tree breeders therefore focus on quantitative traits controlled by several genes [3]. These constraints are associated with the late flowering, slow maturation, long reproductive cycles, and complex mating systems (including self-incompatibility and a high degree of heterozygosity) in trees. Difficulties in identifying the best parents (and controlling their mating), maintaining genetic gain with high heterozygosity [4], and understanding the complex genomes of many tree species causes problems for tree breeders. Genetic modification (GM), on the other hand theoretically allows modification of most individual traits in selected genotypes. Hence, GM technology is much more specific than classical breeding and it can accelerate and allow new strategies for breeding [5].

One main aim with genetic engineering of trees is to produce plants that are resistant to various types of pests [6], [7]. Tree pests can severely effect growth and survival of forest trees and thus inflict large economic losses [8]. A warmer climate in the future could increase these problems [8], [9]. The most common transformations for pest resistance involve the use of *Bacillus thuringiensis* (Bt) genes, enabling the plant to produce Cry toxins lethal to certain targeted insect pests. However, there are considerable risks for the evolution of pest resistance in wild populations that needs to be evaluated and minimized [10], [11]. The Bt toxin leads to cell damage in the insect mid-gut (for more information see [12]). More than 150 different Cry proteins have been identified [12], with examples including Cry3Aa proteins targeting coleopteran insects and the cry1 and cry2 families effective against lepidopteran species [13], [14]. The effectiveness of these toxins against specific pest species on trees has been shown repeatedly in the laboratory [6], [15] and in the field [13]. Still, it is not clear to what degree Bt resistance also will translate into increase tree productivity. Establishing if, and to what degree, plant benefits from the Bt gene with respect to production is essential for cost-benefit analyses of Bt trees, which is the focus of our study.

It has been shown that the production of natural plant defenses are often associated with costs, i.e. there may be a trade-offs between growth and defense [16]–[20]. It has sometimes proved difficult to demonstrate the costs of defensive compounds and such trade-off might also be transient or context-dependent [21]–[23]. If such trade-off also should apply to trees producing Bt toxins is not clear at this point but if such costs exist the realized benefits with Bt resistance is likely context-dependent, i.e. influenced by herbivore levels.

We established an experiment with potted Bt-expressing aspens (*Populus tremula*×*P. tremuloides*) in the greenhouse to explore how experimentally controlled levels of a presumably targeted leaf beetle *Phratora vitellinae* (Coleoptera; Chrysomelidae) affected leaf damage severity and performance of the plants. We hypothesized that GM aspens producing Bt toxins should suffer less damage by the leaf beetle than the isogenic wildtype (Wt) and that survival of the leaf beetle, *P. vitellinae,* would be reduced on Bt aspens compared to the wildtype. In line with the above predictions, we further hypothesized that reduced herbivory would translate into increased growth in Bt aspens compared to the wildtype in the presence of the *P. vitellinae*.

## Methods

### Plant material

We used three isogenic lines of an aspen hybrid (*P. tremula*×*P. tremuloides*) (INRA # 353-38) in which two lines were genetically modified to express Bt toxins, and one was a unmodified line considered a wildtype control (Wt) line. The two genetically modified lines are the Bt17 and Bt27 lines previously described by Genissel et al. [6] and are modified to express a *cry*3Aa Bt- protein targeting Coleopteran species. Bt17 and Bt27 produces toxins in concentrations of approximately 0.05% and 0.0025% of total soluble proteins in the leaves, respectively, and both lines have shown high resistance to the leaf beetle *Chrysomela tremulae* [6].

Plantlets of all lines were propagated in the lab and subsequently planted in 3 L pots in commercially available soil in the green house. During the first 10 days of the establishment phase the plants were covered by individual micro-greenhouses using transparent plastic bags. After removal of the micro-greenhouses, the plants were left an additional 14 days before the experiments started. During the experiment the plants received a commercially available NP-fertilizer (Weibulls “Rika S”) and water was added to the plants when required.

### Experimental design

We used a randomized block design with three plants in each block and a total of 30 blocks. Plants within a block consisted of one individual from each line (Wt, Bt17 and Bt 27) and each block was randomly assigned to the different herbivore treatments (see details below). At the start of the experiment, individual plants (approximately 28.4 cm (SE = 0.3) in height at the time), were covered with a tent of fibre cloth. The cloth was commercially available and is used in agricultural practice to mechanically reduce damage by insect pests. Tents were 1.5 m tall to allow maximum tree growth.

Adult *Phratora vitellinae* (Coleoptera; Chrysomelidae) individuals were collected in the field and to minimize the variation in plant responses due to variations in beetle life history state (e.g. sex and age etc.) the beetles were randomly assigned to different plants and density treatments. Furthermore, the beetles were collected from the same site at the same time (i.e. they belonged to the same generation). Thus, although variation in sex and age among the beetles used in the experiments might have resulted in increased variation in damage levels, the randomization of beetles to different treatments and the large number of beetles used should have minimized this influence. This beetle species is a common herbivore on both willow and aspen species [24] and converts salicyl glucosides from the host plant into a larval defensive secretion which consists mainly of salicylaldehyde [25], [26]. A related beetle species has been shown to be attracted to highly defended trees, where their sequestration of defenses makes them better defended against predaceous ants [27]. Herbivore treatments consisted of no (0 adults), low (3 adults), and high (7 adults) herbivore loads.

### Response measurements

After 5 weeks the experiment was terminated and we counted the number of live adult beetles and larvae (no larvae were introduced to the plants, but some adults were reproductively successful during our experiment). We also measured leaf damage and height, stem, leaf and root biomass of the trees (see details below). For leaf damage, every leaf was assessed for percent damage using a scale with 5% intervals (i.e. 0 equals 0, 1–5 equals 5%, 6–10 equals 10%, and so forth). In addition, each individual plant was destructively harvested and divided into stem, leaf and root parts. To isolate root material the soil was gently removed with a hand shower. The plant fractions were dried to constant mass in a dryer at 40°C.

### Statistical analyses

We used two-way analyses of variance (ANOVA) to determine the effect of herbivore treatment (n = 3), aspen line (n = 3), and their interaction on plant height, dry mass, beetle survival and leaf damage. Plant height at the start of the experiment was used as a covariate in all analyses. When significant effects were shown, subsequent pair-wise comparisons (Tukey's HSD) were used to identify differences between herbivore treatment or aspen line. In addition, when significant interactions occurred, we examined the effect of each factor at each level of the other factor, using simple contrast which test relationships among cell means [28]. In all of the analyses outlined above, the assumptions of ANOVA were tested with residual plots and in cases of heterogeneous variances the data were log(x+1) transformed prior to analysis. All statistical analyzes were performed in SYSTAT 13 [29].

## Results

### Insect survival

**Figure 1:**
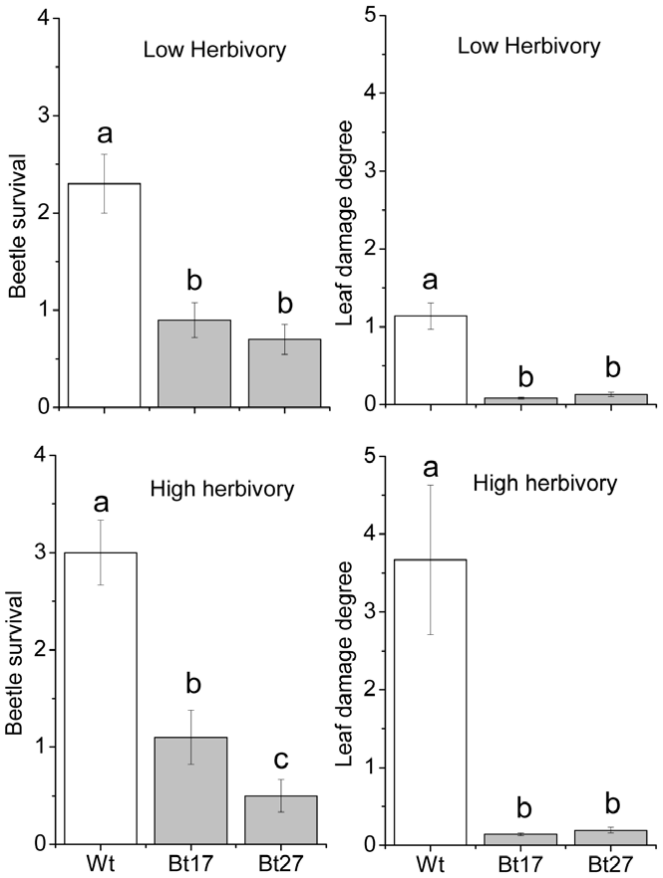
Beetle survival and degree of leaf damage. The mean number of live *Phratora vitellinae* adults per plant and the degree of leaf damage on leaves from Wt, Bt17and Bt27 plants at the end of the trials in the high (initially 7 beetles plant) and low (initially 3 beetles per plant) herbivore density treatments. Bars with different letters indicate significant differences among lines (*P*<0.05).

Herbivore treatment, aspen line and the interaction between these two factors had a significant effect on the survival of *P. vitellinae* adults (F_3,80_ = 49.12, *P*<0.001, F_3,80_ = 38.12, *P*<0.001, F_3,80_ = 10.68, *P*<0.001, respectively). Further analyses of the interaction term revealed that beetle survival was significantly higher on Wt plants compared to the Bt lines in both the high and low herbivore treatment (*P*<0.001 in all cases, Fig. 1). Survival was also significantly lower on line Bt27 than on Bt17 in the high herbivore treatment (*P* = 0.035). The initial height of the plant at the start of the experiment had no effect on beetle survival (*P* = 0.941). Importantly, adult beetles successfully reproduced on Wt plants, but did not reproduce on Bt lines; on average, 3.1 (±2.5) live larvae were found on Wt plants in the high herbivore treatment, but, no live larvae were found on any of the Bt lines.

### Degree of damage

Leaf damage was significantly influenced by herbivore treatment, aspen line and the interaction between these two factors (F_3,80_ = 41.57, *P*<0.001, F_3,80_ = 64.945, *P*<0.001, F_3,80_ = 19.911, *P*<0.001, respectively). In both the low and high herbivore treatments, the damage was significantly higher on the Wt than on Bt17 or Bt27 lines (*P*<0.001, in all cases, Fig. 1). Leaf damage did not differ significantly between Bt lines in any of the herbivore treatments (*P* = 0.758 and *P* = 0.904). Finally, leaf damage was unaffected by the initial height of the plant at the start of the experiment (F_3,80_ = 2.780, *P* = 0.099).

### Plant height

**Figure 2:**
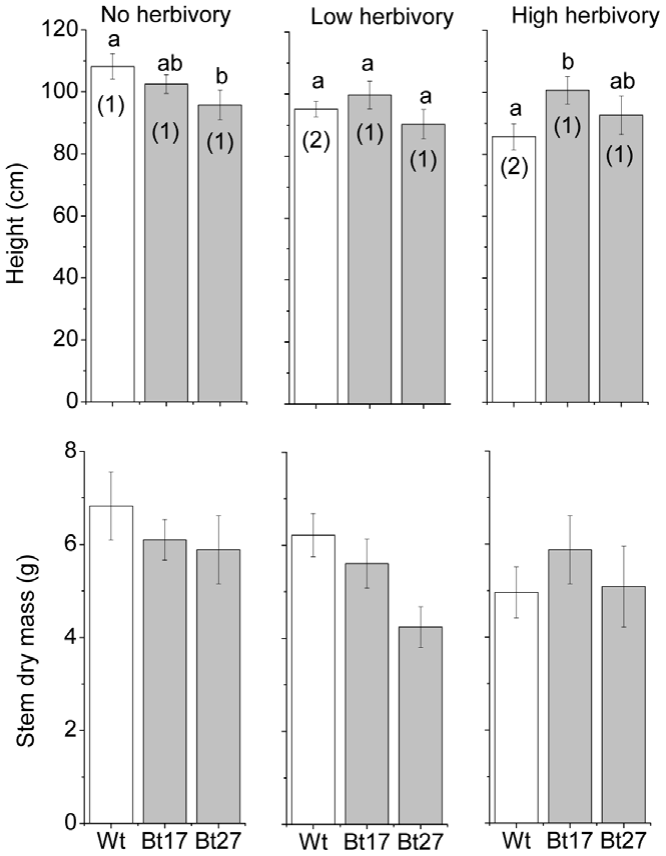
Changes in plant height and stem mass. Mean height and stem mass (and ±SE) of plants from Wt, Bt17and Bt27 lines at the end of the experiment. Bars with different letters indicate significant differences (*P*<0.05) among lines within the same herbivory treatment and different numbers inside the bars denote significant differences within the same line but between treatments. Please note that the ANOVA analysis revealed no significant interaction between line and herbivory for stem mass. As a result, no pair-wise statistical comparisons were conducted for stem mass and the bars therefore lack letters.

Aspen line, herbivore treatment and their interaction all significantly affected plant height, although the effect of herbivory was marginal (F_3,80_ = 4.490, *P* = 0.014, F_3,80_ = 3.078, *P*<0.052, F_3,80_ = 2.597, *P*<0.042, respectively). Within herbivore treatments, trees from the Wt line were significantly taller than the Bt27 line, but not the Bt17 line in the no herbivory treatment (*P* = 0.008 and *P* = 0.216). In contrast, the Wt line was shorter than the Bt17 line but not the Bt27 line in the high herbivore treatment (*P* = 0.006 and *P* = 0.364; Fig. 2). No significant differences in plant height were found in the low herbivory treatment or between the Bt lines regardless of herbivore treatment (*P*>0.05 in all cases). Between herbivore treatments, trees from the Wt line were significantly taller in the no herbivory treatment than in the low and high herbivore treatments (*P* = 0.018 and *P*<0.001), but Wt plants in low and high herbivore treatments did not differ in height (*P*>0.112). Furthermore, the height of the Bt lines did not differ between herbivore treatments *P*>0.05 in all cases). Initial height of the plant at the start of the experiment had a significant effect on final height (*P* = 0.001), but these differences were used as a covariate in all other analyses and were thus accounted for.

### Plant mass

**Figure 3:**
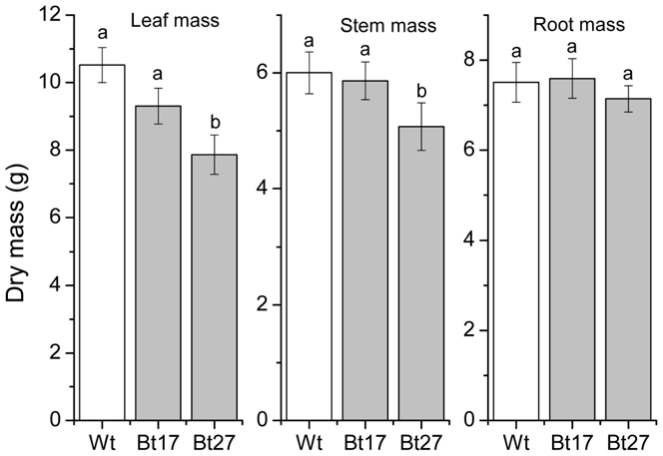
Differences in plant mass between the aspen lines. Mean leaf, stem and root mass (and ±SE) of plants from Wt, Bt17and Bt27 lines), pooled for all herbivore treatments, at the end of the experiment. Bars with different letters indicate significant differences among lines (*P*<0.05).

The dry mass of stems and leaves differed significantly among the lines (F_3,80_ = 4,526, *P* = 0.014; F_3,80_ = 8,576, *P*<0.001, respectively) but there were no significant herbivory treatment or interaction effects (F_3,80_ = 2,036, *P* = 0.137; F_3,80_ = 0,314, *P* = 0.562 and F_3,80_ = 0,314, *P* = 0.731; F_3,80_ = 0,407, *P* = 0.803, respectively; Fig. 2). Overall stem and leaf mass was significantly higher in the Wt and Bt 17 lines than the Bt 27 line (*P* = 0.036, *P* = 0023 and *P*<0.001, *P* = 0.021, respectively; Fig. 3.). There was no significant effect of aspen line or herbivory treatment on root mass, but the initial height of the plant was significantly related to root mass as well as both stem and leaf mass (*P* = 0.013, *P* = 0.002, *P* = 0.020).

## Discussion

We found support for our first hypothesis; the Bt aspens negatively affected survival, growth and reproduction of *P. vitellinae*. Further, the degree of leaf damage inflicted by the leaf beetles was clearly lower on Bt lines than on the Wt line. An earlier study by [6] showed that these trees are highly resistant to the leaf beetle, *Crysomela tremulae,* and we found a similar pattern for the related species, *P. vitellinae*. Field experiments with Bt aspens also suggest a high efficiency against target herbivores [13], [30]. This is promising as it suggests that the Bt effects are consistent across a wider range of field or greenhouse habitats. In fact, the degree of leaf damage in our experiment was so low on Bt plants in both herbivore treatments that they did not differ significantly from the no herbivore control (*P*>0.165 in all cases).

Although earlier field experiments and lab experiment do suggest a high efficiency against target herbivores [13], [30] these studies did not deal with realized benefits in tree growth. Our greenhouse experiment made it possible to address the question of realized benefits under controlled levels of herbivory and has the advantage that the variation in other confounding factors can be kept to a minimum. In support of our second hypothesis, we found indications that increased herbivore resistance also resulted in growth advantages. Intensified herbivory reduced the relative height of the Wt line compared to one Bt line in the high herbivory treatment. In addition, Wt plants were taller in the no herbivore treatment than in both the herbivore treatments. At the same time we showed that this benefit was context dependent, i.e. depended on the degree of herbivory. We failed to detect any significant differences among aspen lines at the low herbivory treatment and the Bt27 line was shorter than the Wt line in the no herbivory treatment.

In contrast to our second hypothesis, the herbivore inflicted differences in height did not translate into significant differences in dry mass production, although the trend was similar to that for plant height (Fig. 2). This could potentially be due to the very high growth potential of the aspens in the greenhouse environment. The plants increased in height from an average of 28 cm to 97 cm during the 5 weeks of the experiment. Good growing conditions (unlimited water, nutrients and light), are known to increase the ability of plants to compensate for herbivore damage [31]–[33]; but see also [34]. The degree of leaf damage was also relatively low with an average of only 3.7% leaf area affected on Wt plants in the high herbivore treatment. This degree of damaged should be compared to estimated levels of insect damages in aspens plantations ranging between 3.8% and ca 50% [35]–[37] and recent field experiments under semi-natural conditions with the same aspen lines which resulted in ∼3.5% leaf damage [38]. Thus, it seems likely that the damage levels, although they did effect height, were too low to have any serious impact on biomass (see also [39]). The plants in our study were also only subjected to herbivory during a relatively short period of time and only during one growing season. Stronger growth responses might have been observed if the plants had been subjected to herbivory for a longer period of time. For example, repeated herbivory is known to reduce the ability of woody plants to compensate for biomass loss due to herbivory [31], [33]. Below ground competition can also reduce compensatory ability in plants [40] but our aspens were grown singly in pots and were therefore not affected by belowground competition. Thus, it is likely that increased herbivore density and repeated herbivory, similar to what can be found in commercial aspen plantations, would lead to detectable growth advantage for Bt aspens. The reduction of plant height of Wt plants but not Bt plants with increased herbivory supported our second hypothesis, but we only found a trend and no significant effects on biomass production. On balance we therefore must conclude that our results only provide partial support for our second hypothesis.

In the absence of herbivores, plants from the Bt27 line actually grow less well than Wt plants. This could suggest that there is a cost associated with the production of Bt toxins. However, line Bt27 produced much less Bt toxins than line Bt 17 (approximately 0.0025% and 0.05%, respectively; [6] and plants from the Bt17 line did not show any reduction in growth compared to the wild type. Thus, the reduction in growth is therefore most likely due to events in the transformation process.

It is well known in plant genetic engineering that many events in the transformation process may cause variability in gene expression or gene silencing and have secondary, unintended effects on plant physiology and fitness [41]–[43]. However, it is currently not possible to determine which of these events that is the most likely cause of the reduced growth of the Bt 27 line or the likelihood that these effects would be manifested under natural growing conditions. In this respect, it is also important to point out that our results should not be considered as representative for Bt plants in general or even all lines of Bt aspens. Our lines were selected due to good performance in the greenhouse but as pointed out above, various factors could influence GM trees physiology and performance. A product-by-product evaluation is always necessary to evaluate both the potential benefits and the potential risks with GM plants [44].

To conclude, in this study we found clear evidence that Bt toxins reduce leaf damage and survival of the target insect herbivore (*P. vitellinae*). The relative increase in height of the Bt17 line compared with the Wt line in the presence of herbivores suggests that this also might translate into increased growth for Bt trees if the herbivore pressure is sufficiently high. Although we were unable to detect significant differences, we found a similar trend for stem biomass as for plant height. We detected no growth response corresponding to the concentrations of Bt toxins produced, suggesting that the indicated growth reduction in one Bt line is more likely due to events in the transformation process and that a hypothesized cost of producing Bt toxins is of subordinate significance.
